# Genomic copy number variations in glaucomatous neurodegeneration

**DOI:** 10.1186/1755-8166-7-S1-I33

**Published:** 2014-01-21

**Authors:** Arijit Mukhopadhyay

**Affiliations:** 1 CSIR-Institute of Genomic and Integrative Biology, New Delhi, India

## 

Copy number variation (CNV) is one of the major factors contributing to genomic diversity and diseases. It has been shown especially for neurodegenerative diseases that CNVs can play a very important role in genetic predisposition of the disease. Glaucoma is a major neurodegenerative disease causing irreversible vision loss across the globe. We wanted to analyze the impact of CNVs in a genome-wide scale in patients of primary open angle glaucoma (POAG) collected from the West Bengal state of India and reproduce our results in another cohort of Caucasian origin. Genome-wide data was generated on 347 POAG cases and 345 controls on Illumina 660W-Quad arrays and CNVs were called using PennCNV. The CNVs were classified as small (<100 kb) and large (>100 kb) and analyzed seprately for their involvement in the disease. A publicly available dataset of POAG cohort of 624 cases and 404 controls from Caucasian origin (GLAUGEN study) was used as a validation cohort and genome-wide CNV data of 208 HAPMAP samples was used as global control. We analyzed genome-wide CNV from 1928 samples. For the large CNVs distribution was significantly skewed toward larger size (>1 Mb) in cases compared to controls and this was replicated in the GLAUGEN data. We found that CNVs >1 Mb are enriched for gene rich regions in POAG patients with 125 genes while for controls a similar percentage of large CNVs overlapped with only 5 genes. In 208 HAPMAP samples CNV >1 Mb overlapped with 95 genes. Interestingly, genes found in the patients were unique and did not overlap with controls or HAPMAP samples. Within CNVs of >1 Mb gene-rich large deletions were ~2 fold enriched in patients compared to duplications irrespective of their ethnic background. Such a bias was not observed in the controls. In the smaller CNV range we performed association analysis and identifed novel regions to be under significantly higher CNV in patients’ comapred to controls. Particularly, a CNV encompassing the transcription factor FOXE3 was significantly enriched in patients of both Indian and Caucasian POAG patietns comapred to their respective controls. A sequence analysis of the gene revealed novel missense mutation in the patients. We have shown that genomic CNVs >1 Mb has significantly higher burden in POAG patient’s genome compared to controls irrespective of the population background. We have also identified candidate genes/regions which are uniquely present in POAG cases and absent in controls from all over the world. Our data provide new insights into role of CNV in pathogenesis of POAG.

